# Burden of intestinal pathogens and associated factors among asymptomatic food handlers in South Ethiopia: emphasis on salmonellosis

**DOI:** 10.1186/s13104-018-3610-4

**Published:** 2018-07-24

**Authors:** Fithamlak Bisetegen Solomon, Fiseha Wadilo Wada, Antehun Alemayehu Anjulo, Hailu Chare Koyra, Efrata Girma Tufa

**Affiliations:** 1 Department of Medical Laboratory, School of Medicine, Wolaita Sodo University, P.O.Box: 138, Wolaita Sodo, Ethiopia; 2 Department of Pharmacy, Wolaita Sodo University, Wolaita Sodo, Ethiopia; 3 School of Public Health, Wolaita Sodo University, Wolaita Sodo, Ethiopia

**Keywords:** Intestinal parasites, *Salmonella*, Sero-group, *S. typhi*, Antibiotic, Multi-drug resistance, Food handlers

## Abstract

**Objective:**

The study aims to assess the burden of intestinal parasites and Salmonellosis among asymptomatic food handlers at meal serving facilities in Sodo town. Antibiotic resistance was also common and increasing among *Salmonella* isolates with multidrug resistance as current concern.

**Result:**

Community based cross-sectional study was carried out from 387 food handlers working in meal serving facilities. Food handlers, 159(41%) had one or more intestinal parasites. *A. lumbricoides* was the most prevalent parasite 30(7.8%), followed by *Taenia* species 26(6.7%) and *Hook worm* 23(5.9%). A total number of 35 *Salmonella* isolates were found of which Sero-group D was the most frequent, 17(48.5%) followed by Sero-group C, 12(34.3%), and B 6(17.1%). Ten (2.5%) isolates were *Salmonella typhi*. Raw meat eating, hand washing after toilet and after touching dirty materials showed significant association with intestinal pathogens. *Salmonella* isolates were highly resistant to ampicillin (85.7%), amoxicillin and tetracycline 74.3% each. Multidrug resistance prevalence of 81.8% was identified. Periodic screening of food handlers is important in order to prevent the transmission of intestinal parasites and Salmonellosis. Treatment needs to be based on accurate laboratory detection to mitigate the spread of drug resistant *Salmonella* strains.

**Electronic supplementary material:**

The online version of this article (10.1186/s13104-018-3610-4) contains supplementary material, which is available to authorized users.

## Introduction

Food-borne infections are common public health problems, which become a significant public health issue all over the world [1]. Food handlers serve as vehicle to transmit food borne illness, during the course of gastrointestinal illness or during and after convalescence depending on the health status of the food handlers, personal hygiene, knowledge and practice of food hygiene [2–4].

About 3.5 billion people are affected by intestinal parasites (IPs), with an estimated 200,000 deaths annually [5]. The most common intestinal helminthes include *Taenia a*, *Hymenolepis*, *Ascaris*, *Strongyloides*, *Trichuris*, *Enterobius vermicularis* and Hook worm [6], and are usually transmitted from contaminated food, water or environment [7]. Potential carriers, food handlers, lead to difficulties in the eradication and control of IPs since they become asymptomatic [8].

*Salmonella typhi* is one of the major causes of food and water borne gastroenteritis in human and causes typhoid fever. It is highly prevalent in countries with poor sanitation during food preparation and poor sewage disposal and handling system [9]. Multi drug resistant (MDR) *S. typhi* has also been increased from time to time and got primary attention in the last few years [10].

Raw beef consumption habit is a potential cause for food borne illnesses in Ethiopia, especially in the study area besides the common factors such as overcrowding, poverty, inadequate sanitary conditions and poor general hygiene [11].

In Ethiopia, provision of safe food has been the primary focus in order to mitigate the spread of food borne outbreaks [3]. Therefore, the main objective of the current study was to determine the prevalence of IPs, with emphasis on *Salmonella* spp. (*S. typhi*) among asymptomatic food handlers.

## Main text

### Methods

#### Study design

A community based cross-sectional study was carried out among food handlers in randomly selected meal serving facilities in Wolaita Sodo town from September 2016 to April 2017. The town is structured in three sub-cities and 11 administrative kebeles. There were 59 meal serving facilities (MSF), restaurants and cafeterias, in the town during the study period. A total number of 5520 food handlers were registered in the town municipality.

The sample size was determined by using a single population proportion formula [10] considering the following assumptions: Z_α/2_ = 1.96 for the standard scale of 95% level of confidence, level of precision = 5%, P = 0.5

$$n = \, {\left( {{Z_{\alpha /2}}} \right)^2}P \, \left( {1 - P} \right)/{d^2} = 384.$$

The total sample size was 422 with 10% non response rate. Since the source population is less than 10,000, correction formula is used to determine the sample size. So the final sample size was *394*.

#### Data collection and analysis

Socio-demographic variables were assessed using an interview with pre-structured questionnaire.

#### Ova/parasite identification

Freshly passed stool samples collected aseptically and examined microscopically following direct wet mount preparations in normal saline, iodine solution and formol-ether concentration sedimentation techniques as per the standards. The parasites identified in any one of the three techniques from a single specimen will be reported as positive.

#### Stool culture

Fecal specimen was homogenized in buffered peptone water (Oxoid, England) and incubated for 24 h at 37 °C. Then, 1 ml aliquot of the enrichment broths was transferred aseptically into 10 ml selinite F broth and incubated at 37 °C for 24 h. After incubation, loop full of colonies were incubated in Xylose lysine desoxycholate agar (XLD) and brilliant green agar (BGA) plates. *Salmonella* presumptive colonies on agar media were subjected onto Triple Sugar Iron agar, Lysine Iron agar, Methyl Red broth, Voges Proskauer broth, Urea broth, Indole test, and Citrate utilization tests and incubated for 24–48 h at 37 °C.

#### Sero-grouping

Sero-grouping of *Salmonella* spp. was done by slide agglutination technique using poly O (AI) and monovalent (O_2_, O_3_, O_4_, O_5_, O_6_, O_7_, O_8_, O_9_, O_15_ and Vi) antigens for identification of *Salmonella* sero-groups, A–E (Difco, Detroit, USA) [11].

#### Agglutination technique

##### Qualitative slide agglutination

A drop of *S. typhi* O and H antigens are added on a drop of serum and rotated at 100RPM and reported as reactive or non reactive by observing agglutination.

##### Semi-quantitative tube agglutination (titration)

Reactive and weakly reactive serum samples were serially diluted by using fresh 0.95% saline preparation from 1:20 to 1:640 for anti O and anti H separately. Then O antigens and H antigens were added in the test tubes and an antibody titer of 1:80 and higher for anti O and 1:160 and higher for anti H antibodies were taken as a cut of value to indicate recent infection of typhoid fever [12].

#### Antimicrobial susceptibility

Antimicrobial susceptibility tests were performed on Muller Hinton Agar (Oxoid, Hampshire, UK) by disc diffusion method. The following antimicrobial agents all from Oxoid were used: ampicillin (10 µg), amoxicillin (10 µg), trimethoprim/sulphamethoxazole (1.25/23.75 µg), amikacin (30 µg), kanamycin (25 µg), chloramphenicol (30 µg), ciprofloxacin (5 µg), ceftriaxone (30 µg), nalidixic acid (30 µg), gentamicin (10 µg) and tetracycline (30 µg). The resistance and sensitivity results were interpreted according to the clinical and laboratory standards institute [13].

MDR was defined as acquired non-susceptibility to at least one agent in three or more antimicrobial categories.

Pan resistance-Resistance for all antibiotics tested.

#### Data analysis procedures

The data was analyzed using statistical package for social science version 21. Bivariate analysis was conducted and variables found to have association with the dependent variable at P value of 0.25 was entered into multiple logistic regression model. The variables P-value less than 0.05 in the multivariate logistic regression were independent factors.

### Results

Out of a total 394 food handling personnel, 387 food handlers participated in the study, giving response rate of 98.2%. Mean age of the study subjects was 25 years (± SD2.8). The median age of the food handlers and their mean work experience was 22 years (± SD4.9) and 3 years (± SD2.1) respectively. Nearly a third (236; 61%) respondents were female and a significant proportion (56.3%) were completed secondary education (Additional file 1: Table S1).

#### Prevalence of intestinal parasites

The study aim to assess common intestinal parasites among food handlers, 159(41%) had one or more IPs and 34(8.8%) food handlers have been diagnosed with mixed IPs. *A. lumbricoides* was the most prevalent parasite 30(7.8%), followed by *Taenia* spp. 26(6.7%) (Additional file 1: Figure S1).

#### Prevalence of salmonellosis

Microbiological culture was done to isolate *Salmonella* species and a total number of 35 *Salmonnella* spp. were found of which Sero-group D was the most frequently isolated 17(48.6%) followed by C, 12(34.3%), and B 6(17.1%). According to widal test, 10(2.5%) of the total isolates were *S. typhi* isolates.

#### Factors associated with intestinal microorganisms

Multivariable logistic regression results showed that raw meat eating habit, hand washing after toilet and hand washing after touching dirty materials have shown significant association (P < 0.05) (Table 1).

**Table 1:** Factors associated with bacterial infection isolated from food handlers (n = 387) in Wolaita Sodo meal serving facilities, 2017

Associated factors	Positivity COR	AOR (95% CI)	P value
Age			
≤ 20	92(23.1%)	3.42(2.51–5.67)	0.0
> 20	306(76.9%)	1	
Work experience (years)			
≤ 2	135(33.9%)	1.52(1.04–3.7)	0.8712
> 2	263(76.1%)	1	
Raw meat eating habit			
Yes	369(92.8%)	3.3(2.4–4.6)	0.001
No	29(7.2%)	1	
Hand wash after toilet			
No	93(23.4%)	2.31(1.34–3.84)	0.0046
Yes	305(76.6%)		
Hand wash after touching dirty materials			
Yes	225(56.5%)	2.4(1.7–3.5)	0.0025
No	173(43.5%)		
Trimmed finger nail			
Yes	95(23.9%)		
No	303(72.1%)	1.78(1.24–2.88)	0.094
Food hygiene training			
Yes	80(20.1%)		
No	318(79.9%)	2.14(1.55–4.91)	0.1206

#### *Salmonella* spp. resistance pattern

According to disk diffusion sensitivity findings, *Salmonella* isolates were highly resistant to ampicillin (85.7%), amoxicillin and tetracycline 74.3% each. Sero-group D showed higher resistance rate to ampicillin, amoxicillin and chloramphenicol with magnitude of 88.2, and 82.4% each for the later two antibiotics (Table 2).

**Table 2:** Antimicrobial resistance pattern of *Salmonella* spp. isolated from food handlers in Wolaita Sodo meal serving facilities (n = 387), 2017

	AMP	TMP–SXT	AMX	K	CIP	CRO	NA	C
*Salmonella* spp. (35)	30(85.7%)	24(68.6%)	26(74.3%)	12(34.3%)	5(14.3%)	10(28.6%)	15(42.9%)	9(27.5%)
Sero-group D (17)	15(88.2%)	11(64.7%)	14(82.4%)	7(41.2%)	3(17.6%)	6(35.3%)	9(52.9%)	5(29.4%)
Sero-group C (12)	10(83.3)	8(66.6%)	8(66.7%)	4(33.3%)	2(16.7%)	3(25%)	4(33.3%)	3(25%)
Sero-group B (6)	5(83.3%)	4(66.7%)	4(66.7%)	1(16.7%)	0(0%)	1(16.7%)	3(50%)	1(16.7%)

*AMP* ampicillin, *TMP–SXT* trimethoprim sulfamethoxazole, *AMX* amoxicillin, *K* kanamycin, *CIP* ciprofloxacin, *CRO* ceftriaxone, *NA* nalidixic acid, *C* chloramphenicol, *CN* gentamicin, *AMK* amikacine, *TTC* tetracycline

#### Antibiogram pattern of multi-drug resistant *Salmonella* spp.

A total of 27(81.8%), MDR *Salmonella* spp. were isolated. Resistance to one or more antimicrobial agents was detected in 33 (91.4%) of the *Salmonella* species, of which two isolates were pan resistant (Table 3).

**Table 3:** Antibiogram pattern of *Salmonella* spp. isolated from 387 food handlers in Wolaita Sodo meal-serving facilities, 2017

Bacterial isolates	Pattern	Antibiotics	No (%)
*Salmonella* spp.	R_0_	None	3(8.6)
n = 35	R_1_	AMX	1(2.9)
	R_2_	TTC, TMP–SXT	2(5.7)
	R_3*_	AMP, TTC, TMP–SXT	3(8.6)
		AMP, TMP–SXT, NA	3(8.6)
		AMP, AMX, NA	3(8.6)
	R_4*_	AMP, TTC, AMX, TMP–SXT	3(8.6)
	R_5*_	AMP, TTC, AMX, NA, CRO, K	3(8.6)
		AMP, TTC, AMX, TMP–SXT, CRO	4(11.4)
		AMP, TTC, AMX, TMP–SXT, K, CN	3(8.6)
	R_6*_	AMP, TTC, AMX, TMP–SXT, NA, CN, AMK, K	2(5.7)
		AMP, TTC, AMX, TMP–SXT, K, CIP	2(5.7)
	R_8*_	AMP, TTC, AMX, TMP–SXT, K, NA, CN, CRO, CIP	1(2.9)
		AMP, TTC, AMX, TMP–SXT, K, NA, CN, AMK, CRO, CIP	2(5.7)

R*: MDR (resistance to more than two class of antibiotic)

### Discussion

The overall prevalence of IPs in the current study, 41% was in harmony with studies conducted at South Ethiopia [14], North East Ethiopia 41.1% [15], Nigeria 38.1% [16], Jimma 44.1% [2] Addis Ababa, 45.3% [17]. On the other hand, lower findings were also reported in the country and elsewhere 14.5% [18], 24.3% [19], 29.1% [4], and 30.5% [8]. Higher prevalence of IPs were reported, in Southeastern Anatolia (52.2%) [20], Abeokuta, Nigeria (97%) [21] and Ethiopia, 63% [18] and 49.4% [1] as compared with the present study. A wide difference in magnitude of IPs across surveys could be due to variation among personal hygiene practices, environmental sanitation and ignorance of health-promotion practices.

*A. lumbricoides* was the leading parasite isolated alone or in combination with other parasites from food handlers in the current study. Similar findings have been reported in previous studies in Ethiopia [2, 4, 14, 15, 17, 22, 23]. Soil transmitted Helminthes, *A. lumbricoides, Taenia* spp., *H. worm* and *S. stercoralis* reported in this study may indicate low personal hygiene in food handlers and the habit of open field defecation of the community.

Even though the magnitude of protozoan’s *G. lamblia* 21(5.4%) and *E. histolytica/dispar* 19(4.9%) is not much higher as compared with intestinal helminthes like *A. lumbricoides* and others, infected food handlers can directly transmit them to consumers if ingested via contaminated food and water. Thus, food handlers should be in a good health and those suffering from diarrhea and dysentery must be excluded from work until they have been completely free of symptoms and must get rest.

*Salmonella* spp. prevalence in this study, 8.8% was in harmony with 6.9% prevalence reported in Arbaminch [24] but higher than study of 5.5% in Abeokuta [21],5% in Haromaya [25] 3.5% in Addis Ababa [17], 3.1% in Gondar [6], 1.6% in Bahir-Dar town, North West Ethiopia [15], and 1% in Mekele [1]. In the contrary to this, higher findings, 13.56% were also reported in Ethiopia [26] and Nigeria, 31.5% [16] and 42.3% [27].

Pooled *Salmonella* prevalence recorded 11.72% of in raw meat in our country [28] could support our finding, even worse the actual data could be higher since our specific setting is one of the region where raw meat consumption is the highest.

The five major sero-groups A, B, C, D, E were reported according to different studies in Ethiopia but the three serotypes B, C and D were the leading groups interchangeably [29–35]. Sero-group D and C were the most frequent sero-groups in the present study of which higher indexes of invasion were also recorded. *Salmonella* sero-group distribution in this study was in agreement with a systematic review of Salmonellosis in Ethiopia from 1974 to 2012 [30] where sero-group D was the most frequent strain [29]. On the other hand, sero-group C occurred more frequently than sero-groups C and B in Central and North Ethiopia. This could be because of large number of *Salmonella* isolates were from children where sero-group C is most common [35], whilst sero-group C ranked first in children, sero-group D was dominant in samples predominantly taken from adults [30].

*Salmonella typhi* as a common aetologic agent to typhoid fever is a public health concern as it was evidenced in north Ethiopia where the episode is the predominant illness among food handlers and street food vendors [36]. In this study, 2.6% *S. typhi* prevalence is comparable with 2.7% reported in Bahirdar University cafeteria [15], but lower than 1.3% in Gondar University cafeteria [3]. The higher percentage of *S. typhi* as compared with previous study could be due to difference in cultural habit of eating raw meat. Comparatively higher, 3.8% in India [37], and much higher findings 8.1% in Hawassa University [38] and 17.4% in Jordan were also documented [39]. Difference in prevalence of *S. typhi* could be attributed to difference in diagnostic technique, different in study settings and recent or previously treated infection. Although a systematic survey on the risk factors is not available, the lower living standard and poor hygienic matters of the general population is suggestive evidence that enteric fever is a threat in present day Ethiopia.

The resistance rates for the isolated *Salmonella* species in this study were high (> 70%) for ampicillin, amoxicillin, and tetracycline. This study was comparable with previous study conducted in Gondar [6] and Central Ethiopia [17]. Our finding was also in line with the pooled proportion of ampicillin (86.1%), and Co-trimoxazole (68%) in Ethiopia [40]. Ceftriaxone resistant *Salmonella* isolates were not revealed in previous studies in Ethiopia [41, 42] in contrary to our study. This may indicate emerging of Ceftriaxone resistance isolates over time. The Magnitude of MDR *Salmonella* spp. in this study corroborated with previous finding in Ethiopia (78.9%) but lower than 100% resistance in Addis Ababa University [17]. The high MDR rate of *Salmonella* isolates and resistance for most of the antibiotics currently used like Ciprofloxacin and Amikacin could limit our antibiotic option for empirical therapy.

Food handling certification, medical checkup, hand washing practice after touching dirty materials and before food preparation have no any significant association with intestinal parasite prevalence which is in line with other studies conducted in the country, north Ethiopia, Bahirdar [15] and South Ethiopia [38] but hand washing practice after toilet have significant association with IPs prevalence which is in harmony with studies conducted in South West [2] and South Ethiopia [24].

### Conclusion

Significant proportion of the population have affected with intestinal parasites and *Salmonella* infection. Considerable number of *Salmonella* isolates showed MDR. *Salmonella* isolates were highly resistant to ampicillin, amoxicillin and tetracycline. Food handlers should be aware of the burden of having IP and salmonellosis through training. Studies on antibiotic resistance should give concern for food handlers. Health education and promotion programs should be sought through extensive training on food hygiene, which would potentially decrease the prevalence of various infections.

## Limitation

Sero-typing of *Salmonella* isolates were not done in this study and observation with inspection team among meal serving facilities wasn’t carried out.

## Additional file

**Additional file 1: Table S1.** Socio demographic and Personal hygiene practice of food handlers (n = 387) Wolaita Sodo town, Southern Ethiopia, 2017. **Figure S1.** Prevalence of intestinal parasites isolated from food handlers (n = 387) in Wolaita Sodo meal serving facilities, 2017.

## Abbreviations

BGA: brilliant green agar
BPW: buffered peptone water
CLSI: clinical and laboratory standards institute
MDR: multi-drug resistant
MSF: meal serving facilities
SNNPR: South nation nationalities and people representative
SD: standard deviation
SPSS: statistical package for social science
XLD: Xylose lysine desoxycholate agar
