# Supplementary material for: Burden of intestinal pathogens and associated factors among asymptomatic food handlers in South Ethiopia: emphasis on salmonellosis
Source: BMC Res Notes. 2018 Jul 24;11:502. doi: 10.1186/s13104-018-3610-4 (PMC6056936; doi:10.1186/s13104-018-3610-4)
Supplement: Supplementary file 1 — Additional file 1: Table S1. Socio demographic and Personal hygiene practice of food handlers (n = 387) Wolaita Sodo town, Southern Ethiopia, 2017. Figure S1. Prevalence of intestinal parasites isolated from food handlers (n = 387) in Wolaita Sodo meal serving facilities, 2017. [file 13104_2018_3610_MOESM1_ESM.docx]

**Table S1: Socio demographic and Personal hygiene practice of food handlers (n=387) Wolaita Sodo town, Southern Ethiopia, 201****7**
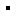


| Socio-demographic characteristics | Frequency | Percent |
| --- | --- | --- |
| Age |  |  |
| < 20 | 72 | 18.6 |
| 21-35 | 262 | 67.7 |
| >35 | 53 | 13.7 |
| Mean | **25** |  |
| Years of work (mean + SD) | 3±2.1 |  |
| ≤2years | 128 | 33.1 |
| >2years  Median | 259  3±1.8 | 76.9 |
| Sex |  |  |
| Male | 158 | 40.8 |
| Female | 229 | 59.2 |
| Education |  |  |
| No formal education | 3 | 0.8 |
| Primary education | 50 | 12.9 |
| Secondary education | 218 | 56.3 |
| Certificate and above | 116 | 30 |
| Ethnicity |  |  |
| Wolaita | 241 | 62.3 |
| Gammo | 19 | 4.9 |
| Gurage | 67 | 17.3 |
| Amhara | 40 | 10.3 |
| other* | 20 | 5.2 |
| Habit of eating raw meat |  |  |
| Yes | 361 | 93.2 |
| No | 26 | 6.7 |
| Hand washing after toilet |  |  |
| No | 90 | 23.3 |
| With water | 217 | 56.1 |
| With Soap and Water | 80 | 20.7 |
| Hand wash after touching dirty materials |  |  |
| No | 219 | 56.6 |
| With water | 137 | 35.4 |
| With Soap and Water | 31 | 8 |
| Use of apron/hair tie |  |  |
| Observed | 63 | 16.3 |
| Not observed | 324 | 83.7 |
| Trimmed finger nails |  |  |
| Yes | 98 | 25.3 |
| No | 289 | 74.6 |
| Food hygiene training |  |  |
| Trained | 80 | 20.7 |
| Not trained | 307 | 80.3 |
| Medical check up |  |  |
| Checked | 110 | 28.4 |
| Not checked | 277 | 71.6 |

**Figure S1:- Prevalence of intestinal parasites isolated from food handlers (n=387) in Wolaita Sodo meal serving facilities, 2017**
